# Rational design for controlled release of Dicer-substrate siRNA harbored in phi29 pRNA-based nanoparticles

**DOI:** 10.1016/j.omtn.2021.07.021

**Published:** 2021-08-08

**Authors:** Daniel W. Binzel, Songchuan Guo, Hongran Yin, Tae Jin Lee, Shujun Liu, Dan Shu, Peixuan Guo

**Affiliations:** 1 Center for RNA Nanobiotechnology and Nanomedicine; Division of Pharmaceutics and Pharmacology, College of Pharmacy; Dorothy M. Davis Heart and Lung Research Institute; James Comprehensive Cancer Center; College of Medicine; The Ohio State University, Columbus, OH 43210, USA; 2 Department of Neurosurgery, McGovern Medical School, The University of Texas Health Science Center at Houston, Houston, TX 77030, USA; 3 The Hormel Institute, Masonic Cancer Center, University of Minnesota, Austin, MN 55912, USA

**Keywords:** RNA nanotechnology, dicer processing, gene regulation, siRNA delivery, exosomes, nanobiotechnology, RNA therapeutics, RNA for cancer therapy

## Abstract

Small interfering RNA (siRNA) for silencing genes and treating disease has been a dream since ranking as a top Breakthrough of the Year in 2002 by *Science*. With the recent FDA approval of four siRNA-based drugs, the potential of RNA therapeutics to become the third milestone in pharmaceutical drug development has become a reality. However, the field of RNA interference (RNAi) therapeutics still faces challenges such as specificity in targeting, intracellular processing, and endosome trapping after targeted delivery. Dicer-substrate siRNAs included onto RNA nanoparticles may be able to overcome these challenges. Here, we show that pRNA-based nanoparticles can be designed to efficiently harbor the Dicer-substrate siRNAs *in vitro* and *in vivo* to the cytosol of tumor cells and release the siRNA. The structure optimization and chemical modification for controlled release of Dicer-substrate siRNAs in tumor cells were also evaluated through molecular beacon analysis. Studies on the length requirement of the overhanging siRNA revealed that at least 23 nucleotides at the dweller’s arm were needed for dicer processing. The above sequence parameters and structure optimization were confirmed in recent studies demonstrating the release of functional Survivin siRNA from the pRNA-based nanoparticles for cancer inhibition in non-small-cell lung, breast, and prostate cancer animal models.

## Graphical abstract

**Figure:**
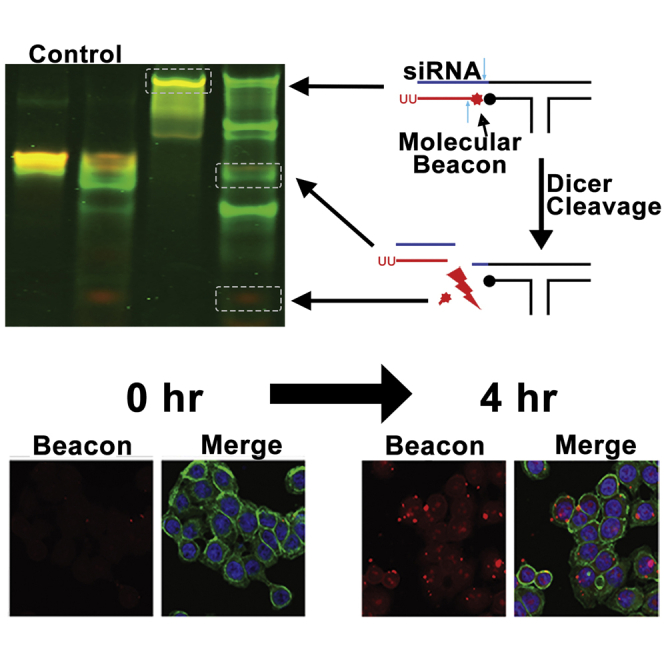

## Teaser

siRNA for silencing genes and treating diseases has been a popular dream but not fully realized. Peixuan Guo and colleagues report the rational design of siRNA to RNA nanoparticles for efficient delivery while avoiding endosomes, thus providing guidelines for siRNA design on RNA nanoparticles to overcome previous roadblocks.

## Introduction

RNA interference (RNAi) is a post-transcriptional gene regulation pathway used by different classes of small RNAs.^1^ Among them, small interfering RNAs (siRNAs)^2^ have attracted attention for their important potentials in drug discovery and development. siRNAs target and bind mRNA to produce gene silencing effects, but their mechanisms are distinct.^3^ siRNA, for its ability to silence genes and potential in treating diseases, has been a popular dream since *Science* ranked this technology as a top ten Breakthrough of the Year in 2002.^4^ siRNA was first discovered in 1998 in *C. elegans* and in mammals in 2001.^5^^,^^6^ Since then, many researchers have worked to bring siRNA to the clinic until the recent FDA approval of Alnylam’s ONPATTRO, the first siRNA drug.^7^^,^^8^ Shortly thereafter, the FDA approved GIVLAARI and OXLUMO, which were also developed and produced by Alnyam.^9^, ^10^, ^11^ Such FDA approvals have moved RNA toward becoming the third milestone in pharmaceutics, following chemical and protein-based therapies.^12^ However, the full potential of siRNA in mainstream therapeutics has not been realized. Further strategies are needed to avoid endosomal trapping.

siRNA is synthetically created, short RNA with matching sequence to its corresponding messenger RNA (mRNA). When internalized into cells, siRNA is incorporated into the RNA-induced silencing complex (RISC), like microRNA (miRNA).^13^ Classical siRNAs are short and typically only 19 bp in length, although longer ones exist; alternatively, longer Dicer-substrate siRNAs range from 20 to 27 bp and require Dicer processing.^14^ Both classical and Dicer-substrate siRNAs interact with and activate RISC. The sense strand of siRNA is degraded,^15^ while the anti-sense strand associated with RISC recognizes its target mRNA for cleavage by AGO2. However, the longer Dicer-substrate siRNAs require Dicer processing before loading into RISC. It is reported that Dicer-substrate RNA duplexes have increased gene silencing activity compared to classic 19-bp siRNAs.^16^

For RNA to be considered for clinical applications it must be stable *in vivo*, thus demanding stability against nucleases. To increase nuclease stability and reduce immunogenicity, nucleotide sugar modifications can be used (most commonly 2′-fluoro [2′-F], 2′-OMethyl, or 2′-O-methoxyethyl [2′-MOE]); however, Dicer-substrate siRNAs do not tolerate sugar modifications at each position, since these modifications can interfere with Dicer recognition. Therefore, careful consideration must be taken to increase siRNA stability. Researchers have created nuclease-stable siRNA while remaining sensitive to Dicer by only modifying key nucleotide sites against nucleases and reducing off-target effects.^17^, ^18^, ^19^, ^20^, ^21^ 2′-F, 2′-OMethyl modifications on the sense strand and at the termini of anti-sense strands retained Dicer processing. Additionally, 2′-MOE modifications were very site specific because of the bulky size of the modification. However, heavy modification of the anti-sense strand resulted in Dicer inactivity. Understanding and exploiting Dicer processing is of significance in the design and optimization of siRNA for efficient gene knockdown.

The Dicer enzyme belongs to the RNase III family, which can cleave long double-strand RNAs (dsRNAs) into small RNAs.^22^ It is reported that the structure of the human Dicer is L shaped and composed of a head, body, and base.^13^^,^^22^^,^^23^ The head is the PAZ (piwi/argonaute/ zwille) domain, which is also the RNA binding domain. The PAZ domain has a high affinity for a 3′ protruding 2-nt overhang on RNAs. Two RNase III domains are located in the “body” and form the processing center, where each RNAase III cleaves one of the long dsRNAs, and the base is an N-terminal DExD/H-box helicase domain with a clamp-shaped structure. Dicer can cleave long dsRNA into 21-22 bp dsRNA. Dicer itself is like a molecular ruler since the distance between the binding PAZ domain and cutting RNase III domain is about the length of 21-22 bp siRNA.^24^^,^^25^ Many factors affecting Dicer processing have been investigated, including mutations to the PAZ domain responsible for RNA recognition and the design of siRNA in relation to the 5′/3′ structuring for Dicer binding and inclusion of chemical modifications (discussed above).^26^, ^27^, ^28^, ^29^ However, some conclusions are inconsistent or contradictory and seem to be specific to each siRNA sequence.

To achieve *in vivo* gene knockdown, another key is efficient delivery of RNAi into cancer cells. Although many nanocarriers have been developed, our studies^12^^,^^30^^,^^31^ have shown that RNA nanotechnology provides one of the best strategies to deliver RNAi into cancer cells.^32^, ^33^, ^34^ By definition, RNA nanotechnology is the bottom-up construction of nanostructures composed primarily of RNA, including the core scaffolding and any functional group attached to the nanoparticle.^34^ Since its conception, more and more complex RNA nanoparticles have been constructed with high thermodynamic stability and proven to function well in *in vivo* applications.^32^^,^^35^, ^36^, ^37^, ^38^ Our RNA nanoparticles, using the phi29 DNA packaging motor packaging RNA (pRNA) as a core motif, are homogeneous in size, structure, and stoichiometry; are thermodynamically and chemically (after 2′-F modifications) stable;^36^^,^^39^, ^40^, ^41^, ^42^ retain authentic folding and independent functionalities of all incorporated modules (RNA aptamer, siRNA, miRNA, or ribozyme);^36^^,^^43^ and are non-toxic^42^^,^^44^ and highly soluble and display favorable biodistribution and PK/PD profiles.^36^^,^^39^^,^^42^^,^^44^, ^45^, ^46^ Furthermore, the application of RNA nanoparticles to exosomes, 30- to 150-nm membranous vesicles of endocytic origin, as a way of intercellular communications^47^^,^^48^ allows for both the loading of RNA nanoparticle-siRNA cargos and displaying tumor-targeting ligands.^30^ Exosomes have an endomembrane-like membrane property (structure, lipid, peptides, protein, etc.) and have been shown to carry genetic materials, especially RNA, as a form of intercellular communication.^47^, ^48^, ^49^, ^50^, ^51^, ^52^, ^53^, ^54^ Exosomes have been considered for their therapeutic applications because of their favorable size and are well tolerated *in vivo*.^51^ Therapeutic payloads, such as siRNA, can remain fully functional after delivery to cells by exosomes.^47^^,^^48^^,^^52^^,^^55^ These exosomes have demonstrated their ability to fuse to the cell membrane of the targeted cells, resulting in the dumping of their cargo directly into the cytosol of cells.^56^ As such, we utilize these exosomes for their ability to avoid endosome trapping and therefore increase the efficiency of delivered siRNAs.^56^

RNA nanotechnology has gained significant interest in the development of novel nanostructures.^57^, ^58^, ^59^, ^60^, ^61^, ^62^, ^63^, ^64^ Several researchers have used these RNA nanoparticles for the inclusion of RNAi components, including siRNAs, and examined their specific gene-silencing abilities.^65^, ^66^, ^67^, ^68^, ^69^, ^70^ Similar to our approaches of applying RNA nanoparticles to exosomes, complex delivery vehicles have been created by functionalizing RNA nanoparticles with polymer nanoparticles^71^ and exosomes^72^ for improved siRNA delivery. Thus RNA nanoparticles are poised to overcome the previous roadblocks in siRNA therapies. However, little is known on how the siRNA delivered by RNA nanoparticles and exosomes interacts with the Dicer complex to achieve efficient knockdown of target genes.

In the present study we examine whether Dicer-substrate siRNA can be incorporated onto pRNA-based RNA nanoparticles and their required design parameters to retain Dicer processing. We integrate and test factors affecting Dicer processing, which directed us to design siRNA-loaded RNA nanoparticles that can function as a Dicer substrate to generate a robust gene regulation tool. Dicer-substrate siRNA is used in these studies because of the ease of inclusion into RNA nanoparticles by simple helical extension, increased efficacy in gene knockdown over short siRNA, and reduced immune responses over short siRNA.^14^^,^^16^ Unlike shorter siRNA, Dicer-substrate siRNA has not translated to the clinic because of delivery issues and requirements of Dicer processing; here we aim to overcome these issues. Besides inclusion of 3′-2-nt overhangs and a 5′-phosphate group, we also investigated whether chemical modifications affect Dicer processing. Based on the screened factors, Luciferase 2 (*LUC2*) siRNA was designed to be incorporated into RNA nanoparticles. To demonstrate the Dicer processing, a molecular beacon was designed and constructed to monitor the activity. Our findings suggest that integration of length, nucleotide components, structure, and RNA nanotechnology into the designs of RNAi achieves efficient silencing of targeted genes in an *in vivo* cancer therapy setting. As such, our findings provide design parameters for siRNA incorporation into RNA nanoparticles for efficient gene silencing.

## Results

### RNA nanoparticles harboring siRNA were processed by Dicer into functional siRNA resulting in cleavage of target gene

**Figure 1:**
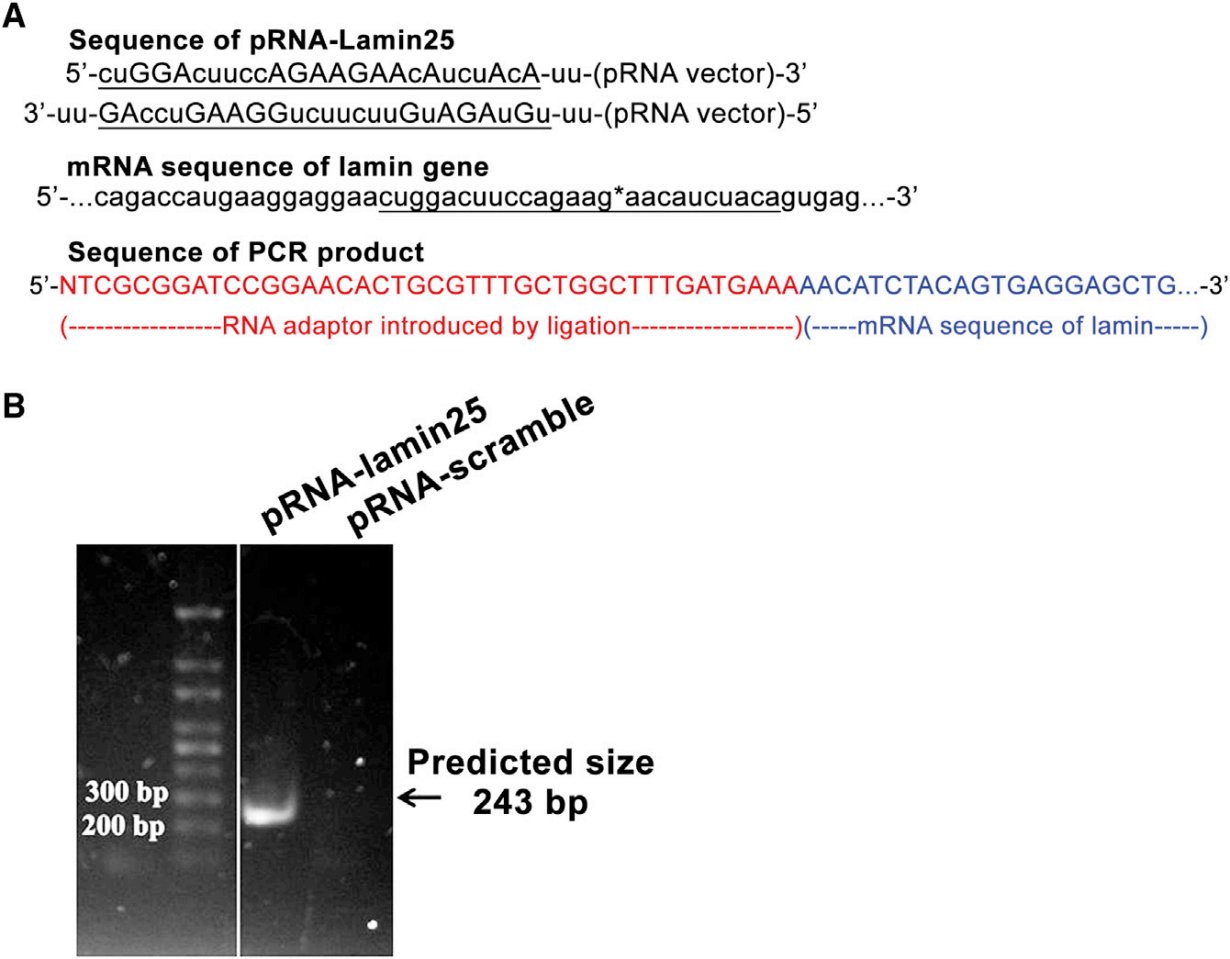
Identification of the specific cleavage site of mRNA delivered by RNA nanoparticles into KB cells (A) Site-specific cleavage of Lamin A/C mRNA induced by pRNA-lamin demonstrated by 5′ RACE. pRNA-lamin25 was constructed by linking a siRNA sequence (underlined) with a pRNA vector via UU linkers at the sense (upper) and antisense (lower) strand. Sequences of Lamin A/C mRNA that are identical with the siRNA are underlined, and the cleavage site is marked with a star. Sequencing results show that the PCR product contains a RNA ligator sequence (red) and part of the Lamin A/C mRNA sequence. (B) PCR products resulting from outer primer pairs were separated in 1.2% agarose gel.

RNA nanoparticles based on the phi29 pRNA were constructed to include Lamin25 siRNA extended on the pRNA 5′/3′ terminal end (pRNA-Lamin25). Lamin A/C was chosen as a model gene to demonstrate the specific cleavage of mRNA by a 5′ rapid amplification of cDNA ends (RACE) technique to prove that the gene-silencing activity of pRNA nanoparticles is mediated by RNA interference.^73^ pRNA-Lamin25 was designed similar to that described by Zhang et al.,^74^ with 25-bp siRNA linked to a pRNA vector by a double uracil (-UU-) linker (Figure 1A). RNA was prepared by *in vitro* transcription by T7 RNA polymerase and transfected into KB cells. The gene-silencing efficiency of pRNA-Lamin25 was first demonstrated by qRT-PCR (Figure S1). A RNA ligation step and two rounds of PCR were used to reveal the sequence of mRNA after cleavage. A DNA fragment representing the cleaved mRNA was detected in the pRNA-Lamin25 sample with the size between 200 bp and 300 bp in gel electrophoresis (Figure 1B). The mRNA cleavage was expected to be 243 bp in length, indicating successfully cleaved Lamin A/C. Sequencing results of the excised DNA fragment confirmed that Lamin A/C mRNA was cleaved at a position 10 nt from the 5′ end of the antisense of Lamin25 siRNA sequence as predicted. No fragment was detected in the control samples treated with pRNAi-scramble (Figure 1B, lane 2). These data demonstrate that gene silencing mediated by the pRNA-siRNA is induced by RNAi pathway as in a standard siRNA duplex. The incorporation of siRNA into RISC and cleaving the Lamin A/C mRNA proves that RNA nanoparticles are able to deliver siRNA sequences into cancer cells and specifically silence its respective gene.

### Development of RNA nanoparticle molecular beacons for observation of *in vitro* processing and release of siRNA

**Figure 2:**
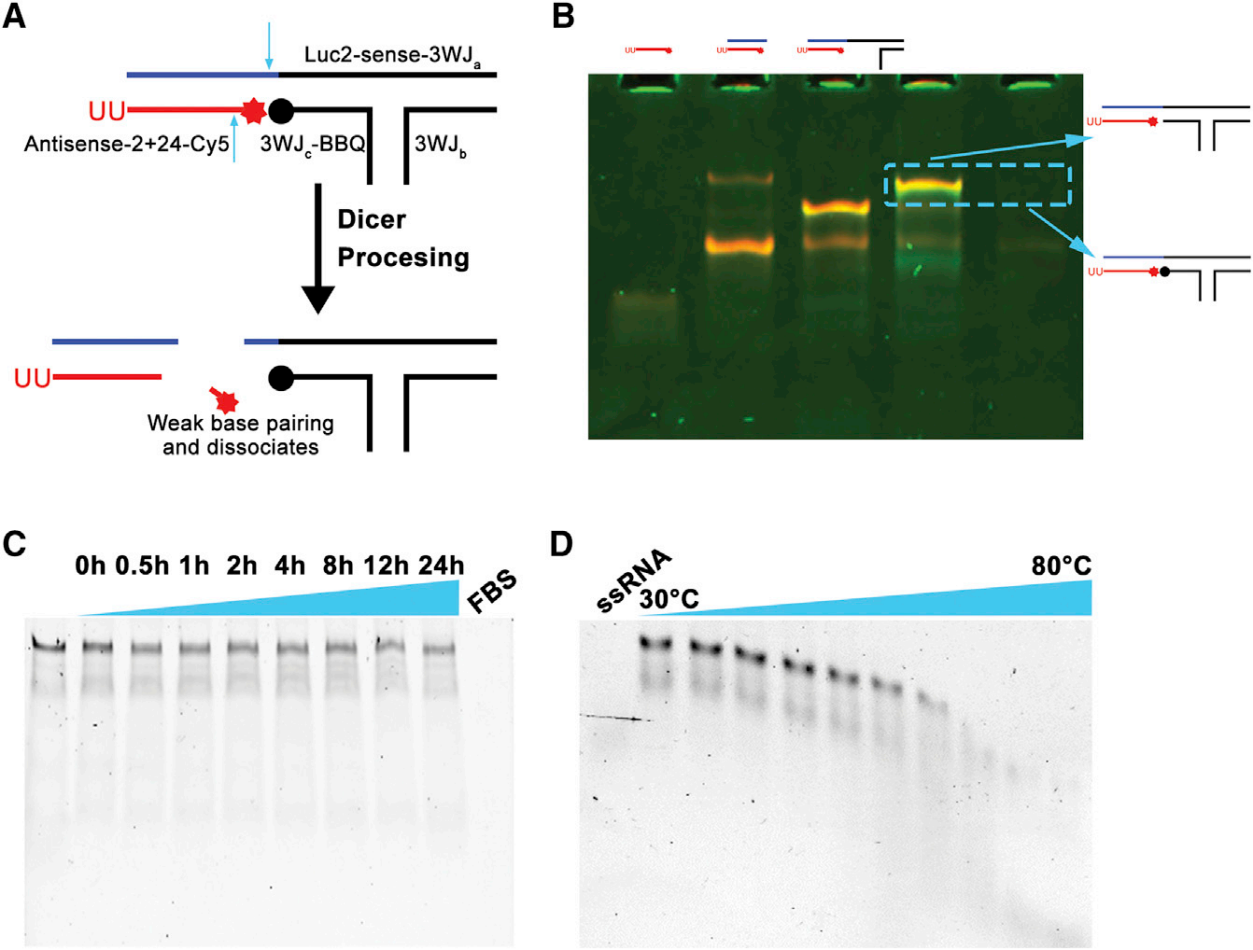
Construction and stability studies of 3WJ/Luc2-siRNA (A). Design of 3WJ/*LUC2*-siRNA molecular beacon (red star: Cy5 fluorophore, black circle: BBQ650 quencher). (B) Assembly of 3WJ/*LUC2*-siRNA molecular beacon assayed by 15% native polyacrylamide gel electrophoresis (PAGE) (green: ethidium bromide [EB], red: Cy5, yellow: overlap). (C and D) Serum stability (C) and thermodynamic stability (D) of 3WJ/*LUC2*-siRNA assayed by 15% native PAGE.

Based on the above results, it was determined that siRNA sequences can release from RNA nanoparticles within the cells. However, confirmation of Dicer processing of siRNA from RNA nanoparticles has not been observed, nor is the release mechanism of siRNA from the RNA nanoparticles understood. pRNA-3WJ RNA nanoparticles were constructed, incorporating 24-bp 3′-UU overhang luciferase siRNA oligo (*LUC2*-siRNA). The pRNA-3WJ motif was used in these studies because the ease of construction and the multivalent nature of the pRNA-3WJ allow for inclusion of siRNA on one helical branch while targeting ligands, such as RNA aptamers, can be added to other branches to fulfill specific and favorable cancer targeting.^36^^,^^44^^,^^45^^,^^75^ This design takes advantage of the high chemical and thermodynamic stability as well as multivalency of the 3WJ scaffold for therapeutics loading and delivery.^38^^,^^76^ To demonstrate that the siRNA incorporated to 3WJ RNA nanoparticles could be processed by Dicer for higher gene regulation efficacy, a molecular beacon was designed accordingly by introducing a fluorophore/quencher pair into the nanoparticles (Figure 2A). A Cy5 fluorophore was conjugated to the 5′ end of antisense strand while a BBQ650 fluorescence quencher was attached to the 3′ end of the 3WJ-c strand adjacent to the Cy5 label. Such design quenches the Cy5-siRNA signal until the siRNA is processed by Dicer, thus creating a short, Cy5-labeled sequence on the 3WJ nanoparticle that is quickly dissociated because of lack of strong base pairing resulting in recovery in fluorescent signal.

Stepwise assembly of the 3WJ/*LUC2*-siRNA was confirmed by 15% polyacrylamide gel in native folding conditions, and incorporation of BBQ650 resulted in high quenching of the Cy5 signal (Figure 2B). Serum stability and temperature gradient gel electrophoresis (TGGE) studies proved resulting nanoparticles that have high chemical and thermodynamic stability, respectively (Figures 2C and 2D), which guarantees the low background noise signal due to lack of *in vivo* degradation of the nanoparticles.

This 3WJ-*LUC2*-siRNA beacon design was used to monitor Dicer processing by intracellular imaging. When the nanoparticle is intact, Cy5 signal is quenched by the BBQ650 because its close proximity and low fluorescence could be detected (“OFF” status). The Cy5 fluorescence was restored when the nanoparticles underwent processing by Dicer and the short fragment with Cy5 fell off of the nanoparticle, creating distance with the BBQ650 (“ON” status) (see Figure 2A). By comparing the fluorescence intensities between 3WJ/*LUC2*-siRNA-Cy5 and 3WJ/*LUC2*-siRNA-Cy5/BBQ650 (molecular beacon), we have a better understanding about the processing of the siRNA in the cell cytosol and its processing efficiency.

### Direct observation of *in vitro* siRNA processing from RNA nanoparticles

Exosomes are lipid vesicles released from cells typically for intercellular communications.^77^ We previously modified the isolated exosomes from HEK293 cells with RNA nanoparticles and demonstrated that RNA-displaying exosomes are able to deliver loaded cargos directly to the cytosol of cells.^30^^,^^56^^,^^78^ HEK293 exosomes were previously shown to have an average size of 96 nm via nanoparticle tracking analysis, a negative zeta potential of −4.6 mV via dynamic light scattering, and expression of TSG101, a typical exosome-specific marker.^30^ With concerns of cytosol trapping, thus preventing siRNA from releasing into the cytosol, we loaded 3WJ/*LUC2*-siRNA beacon nanoparticles into exosomes to be delivered to HT29 cells with continuous luciferase expression (HT29-luc). Additionally, the exosomes were decorated with previously developed RNA nanoparticles harboring folate (FA) to provide specific targeting and binding to the FA receptor-expressing HT29 cells.^30^ Confocal fluorescent microscopy and flow cytometry (fluorescence-activated cell sorting [FACS]) demonstrated high binding efficiency of FA-targeting exosomes (FA/EV/*LUC2*-siRNA/Cy5) to the HT29-luc cells (Figure S2A) and internalization of the 3WJ/*LUC2*-siRNA/Cy5 loaded into the exosomes over controls (Figure S2B). A clear shift in peak fluorescence of the HT29 cells was seen in both FACS and confocal studies of the FA/EV/*LUC2*-siRNA/Cy5 over the 3WJ/EV/*LUC2*-siRNA/cy5, with nearly triple the fluorescence intensity seen in the FACS results.

**Figure 3:**
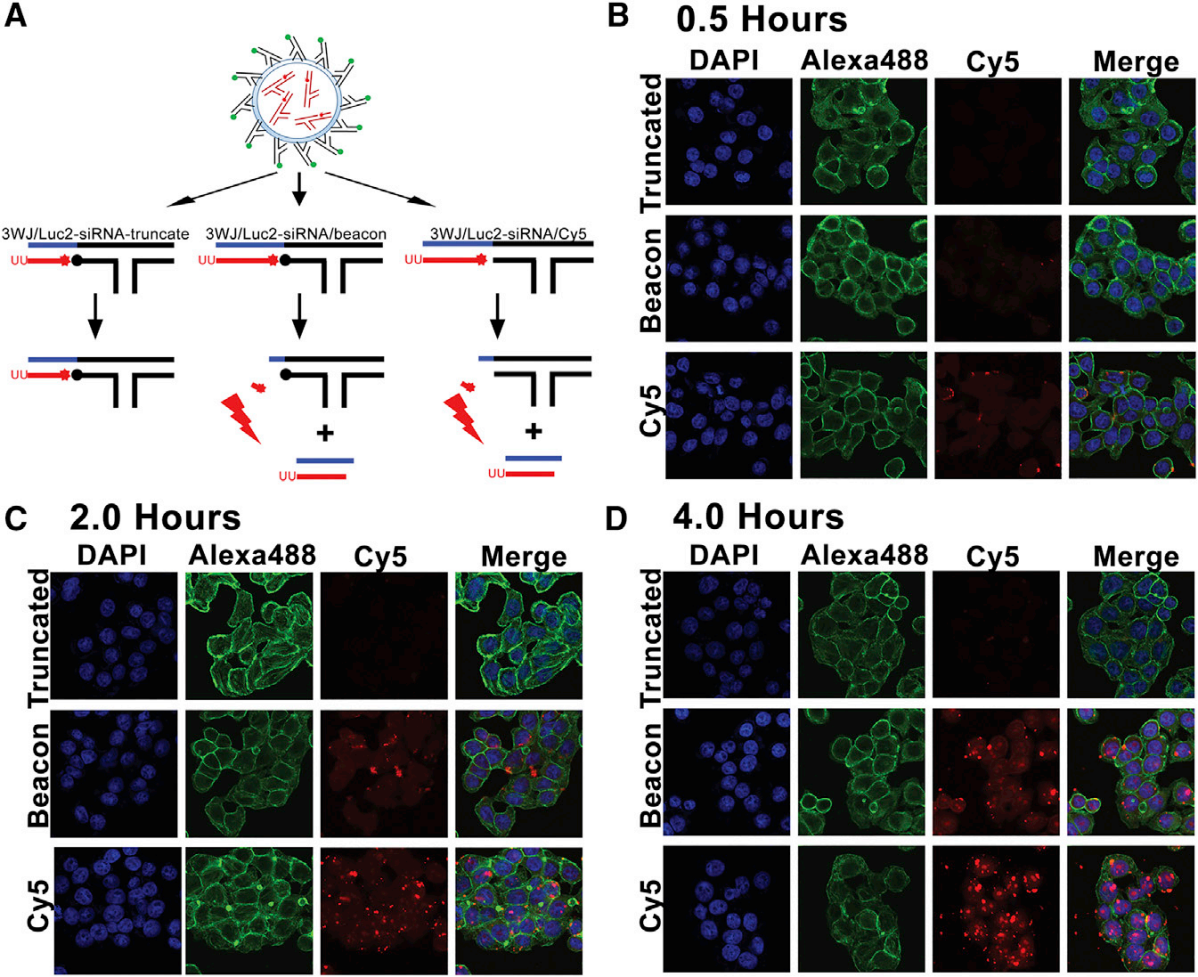
Experimental design for intracellular imaging studies (A) Folate exosome designs carrying RNA nanoparticle beacons (3WJ/*LUC2*-siRNA-truncated beacon, 3WJ/*LUC2*-siRNA beacon, 3WJ/*LUC2*-siRNA/Cy5); red star: Cy5 fluorophore, black circle: BBQ650 quencher. (B) Intracellular imaging of Dicer processing of 3WJ/*LUC2*-siRNA delivered by exosomes after 0.5-h incubation with HT29 cells. (C) Intracellular imaging of Dicer processing of 3WJ/*LUC2*-siRNA delivered by exosomes after 2-h incubation with HT29 cells. (D) Intracellular imaging of Dicer processing of 3WJ/*LUC2*-siRNA delivered by exosomes after 4-h incubation with HT29 cells. Blue: nuclei, green: cytoskeleton, red: RNA.

Utilizing the exosome delivery vehicle allowed the 3WJ/*LUC2*-siRNA beacon nanoparticles to be efficiently distributed to the cytosol of the cancer cells for a time course study of siRNA processing by Dicer. As a positive control to demonstrate Dicer processing rather than random RNase degradation, a truncated 19-bp *LUC2*-siRNA was placed onto the 3WJ (S1: 3WJ/*LUC2*-siRNA-tru) (Figure 3A). 3WJ/*LUC2*-siRNA/Cy5 (S3) nanoparticles were also tested as a total Cy5 signal control. 3WJ/*LUC2*-siRNA beacon (S2) as well as control nanoparticles were delivered by the FA-displaying exosomes. The processing of siRNA from 3WJ scaffold was reflected by Cy5 fluorescence gradual increase in signal over time, as shown in confocal microscopy studies (Figures 3B–3D). As such, the data directly demonstrated that Dicer is able to bind and cleave siRNA incorporated into RNA nanoparticles. This proved that the RNA nanoparticle motif does not interfere with Dicer siRNA processing due to steric hindrance or processing issues as a result of the nanoparticles’ branched nature. It is noted that fluorescence intensity in the 3WJ/*LUC2*-siRNA/Cy5 positive control increases over time because of the longer incubation time of the RNA nanoparticle/exosome complex with cells. This allows for increased levels of 3WJ/*LUC2*-siRNA/Cy5 to be released in the cells, resulting in higher fluorescence levels over time. However, such control serves as a siRNA delivery vehicle control to ensure that siRNA is being delivered within cells.

### *In vitro* identification of the specific cleavage site of siRNA by Dicer as RNA nanoparticle design principle

**Figure 4:**
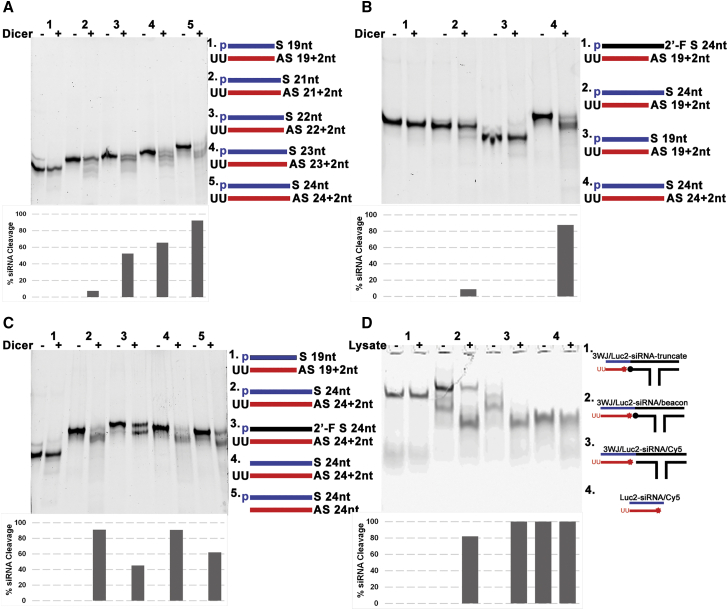
The impact of siRNA length and chemical modifications on Dicer processing (A) *In vitro* Dicer processing of DS/*LUC2*-siRNA in different lengths (19, 21, 22, 23, 24 bp). p, phosphate group; S, sense strand; AS, antisense strand. (B and C) *In vitro* Dicer processing of different designs of DS/*LUC2*-siRNA. Each sample was run as siRNA control without Dicer (−) and with Dicer (+). (D) *In vitro* processing of siRNA from 3WJ RNA nanoparticles when incubated with cell lysates. The treated siRNAs were separated in 1.2% agarose gels in (A–C) and in (D) separated in 15% native PAGE. All siRNA cleavage was calculated by gel band quantified by ImageJ.

In order to better understand the Dicer processing of siRNA from our RNA nanoparticles and provide design principles for siRNA incorporation onto RNA nanoparticles, we examined Dicer processing to various siRNAs *in vitro*. Rational design of siRNA should be illustrated when considering incorporation to RNA nanoparticles to ensure the intracellular Dicer processing of siRNA. Therefore, different factors affecting Dicer processing including siRNA length, 5′-phosphate, 3′-2-nt overhang, and 2′-F modifications were tested based on the Luciferase 2-siRNA used above. The base pairing length is a key factor, as Dicer processes siRNA according to the loop-counting rule.^79^ dsRNA substrate is anchored to the PAZ domain, and the specified distance between the anchored helical end of the dsRNA and the RNase active site in the RNaseIII domains serves as a molecular ruler.^2^^,^^27^^,^^80^ To test the cleavage based on the loop-counting rule, different lengths (19 bp, 21 bp, 22 bp, 23 bp, and 24 bp) of *LUC2*-siRNAs were designed and constructed (Table S1) to be tested for Dicer processing using Human Recombinant Dicer Enzyme. After incubation with the Dicer enzyme, 3WJ-siRNA conjugates were assayed by polyacrylamide gel, looking for a cleaved product. The results demonstrated that siRNA longer than 21 bp showed obvious processing, and 24-bp *LUC2*-siRNA could be processed with the highest efficiency (Figure 4A). Shortening either strand of the double-stranded *LUC2*-siRNAs resulted in inhibition of Dicer processing (Figure 4B), which is due to the dimer structure of the RNaseIII domain. In addition, siRNA constructs regarding 5′-phosphate, 3′-UU overhang, and 2′-F modification were tested for Dicer processing (Figure 4C). 2′-F modifications were selected, as they are well studied in our pRNA-3WJ system and have been shown not to affect the 3WJ structuring while increasing thermodynamic and enzymatic stabilities.^38^^,^^76^ Quantification of Dicer-processed bands demonstrated that the absence of 3′-UU reduces Dicer processing efficiency to only 62% (Figure 4C, lane 5), which may be due to less favorable Dicer enzyme binding. When including enzyme-stabilizing chemical modifications, 2′-F modifications to the pyrimidines of the siRNA sense strand also decreased the processing to 45% (Figure 4C, lane 3); however, the presence of a 5′-phosphate to the sense strand did not affect Dicer processing much in our testing (Figure 4C, lanes 2 and 4). While the decreased Dicer processing in adding 2′-F modifications is disappointing, these modifications are typically required for *in vivo* applications for the increased thermodynamic and enzymatic stabilities of the RNA nanoparticles. However, the incorporation of the 3′-UU overhang of the anti-sense strand significantly improves Dicer processing.

From our testing we were able to further demonstrate that loading of siRNA onto RNA nanoparticles still allows for Dicer recognition and processing of the siRNA (Figure 4D). The use of the molecular beacon demonstrated that 24-bp siRNA is cleaved from the nanoparticle, resulting in Cy5 signal. Dicer processing of various siRNA designs indicated that when incorporating siRNA onto RNA nanoparticles, the length of the siRNA must remain at a minimum length of 22 bp.

### Functional siRNA releasing from the RNA nanoparticles has been confirmed in multiple animal trials on lung, breast, and prostate cancer models

**Figure 5:**
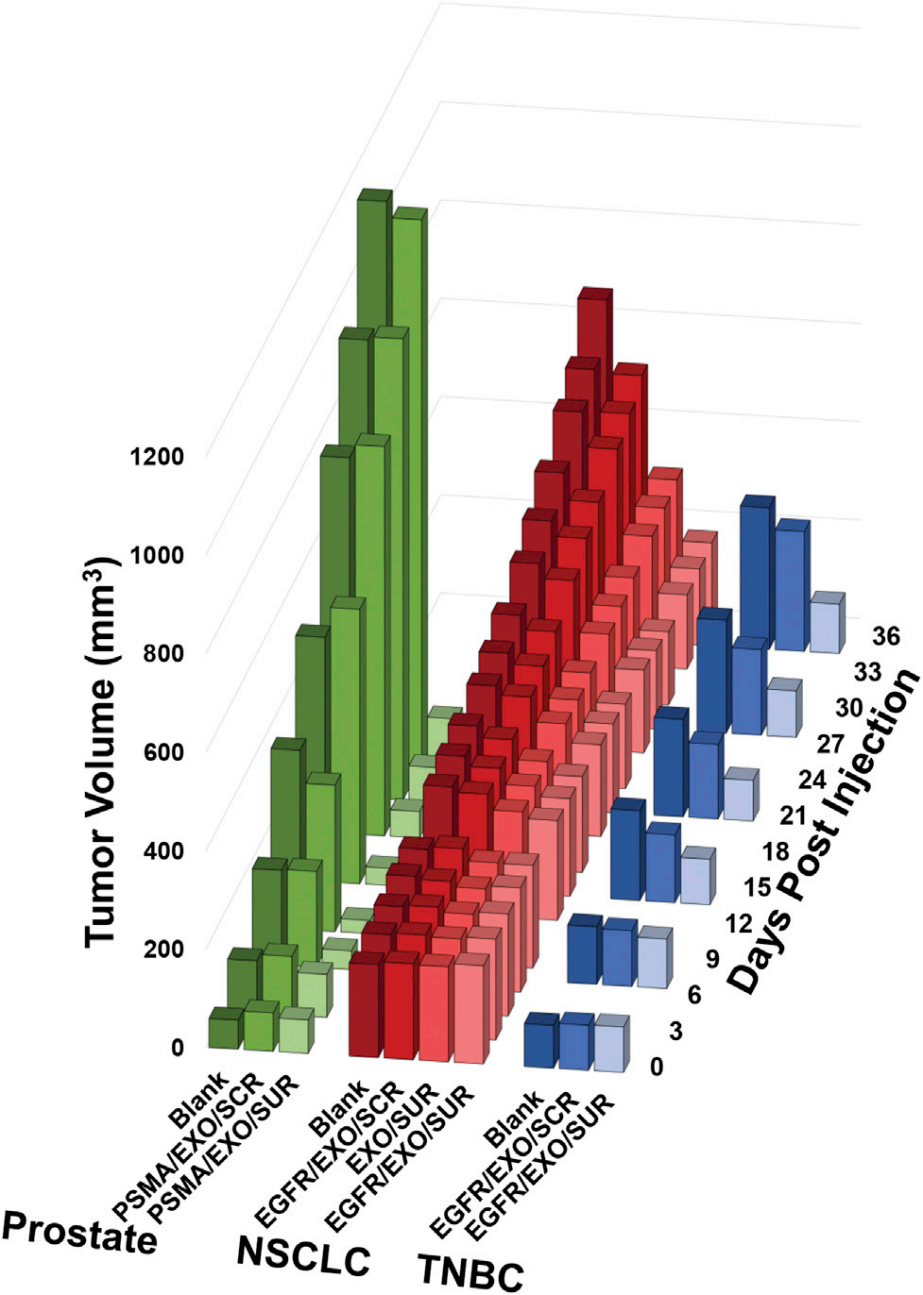
Summary of previous animal trials to elucidate the processing and releasing of Survivin siRNA incorporated in the RNA nanoparticles The data is from models of non-small-cell lung,^82^ breast,^30^ and prostate^30^ cancer. RNA nanoparticles harboring Survivin siRNA were loaded into exosomes and decorated with either epidermal growth factor (EGFR) or prostate-specific membrane antigen (PSMA) RNA aptamers and delivered to prostate cancer, triple negative breast cancer (TNBC), and non-small-cell lung cancer (NSCLC) tumors in mice. Repeated administration demonstrated strong tumor inhibition over control nanoparticles. The data in this figure are a summary of previously published work. Inclusion of the previously published animal data follows the journal policy of *Molecular Therapy Nucleic Acids*, with figure copyright permission from the publishers and original data from the authors.^30^^,^^81^

Furthermore, our group has proven the *in vivo* delivery of siRNA via RNA nanoparticles harboring Survivin siRNA loaded into exosomes (Figure 5).^30^^,^^81^ Microscopy studies demonstrated that the exosomes allow for membrane fusion and cytosol dumping of therapeutic cargos,^56^ while resulting in ultra-high inhibition of tumor growth in *in vivo* tumor models.^30^ While it is thought that endosome entrapment can allow for the slow release of stable siRNA into the cells,^82^ our exosomes avoid endosome entrapment and create direct siRNA delivery to the cytosol of targeted cells for swift treatment.^56^

The releasing of functional siRNA from our RNA nanoparticles was documented by animal trials in multiple publications showing the inhibition of tumors in non-small-cell lung (NSCLC),^81^ triple negative breast (TNBC),^30^ and prostate^30^ cancer models when Survivin siRNA was fused onto RNA nanoparticles and delivered to tumors (Figure 5). Prostate-specific membrane antigen (PSMA) RNA aptamers allowed for specific targeting and delivery of the siRNA to PSMA+ prostate xenograft tumors in nude mice using LNCaP-LN3 cells.^30^ Additionally, epidermal growth factor (EGFR) RNA aptamers were displayed on the surface of exosomes for delivery to orthotopically developed TNBC tumors using MDA-MB-468 cells^30^ and, separately, NSCLC xenograft tumors using H596 cells.^81^ Exosomes were delivered intravenously via tail vein injection. Figure 5 demonstrates that, across three tumor models, exosomes modified with RNA nanoparticles are able to deliver Survivin siRNA that is then processed in the tumors, resulting in strong tumor inhibition. Full data on these studies can be found in Pi et al.^30^ and Li et al.^81^ Animal studies were conducted in accordance with an approved protocol adhering to the Institutional Animal Care and Use Committee (IACUC) policies and procedures at The Ohio State University.

## Discussion

Since its discovery in 1998,^5^ RNAi sequences have shown great promise in treating diseases and cancers by silencing specific responsible genes. Many large pharmaceutical companies and research groups have invested billions of dollars toward developing siRNA and other RNAi technologies for the treatment of cancers.^83^, ^84^, ^85^, ^86^, ^87^ However, difficulties in producing a safe and efficient delivery system for the siRNA resulted in diminishing interest. Yet the promise of siRNA for the treatment of cancers and viral infections still remains.^88^ There is a desperate need for more efficient delivery vehicles that not only specifically target disease sites with high affinity but also deliver the RNAi payloads for Dicer processing.

In the presented study, the pRNA-based RNA nanoparticles resulted in specific knockdown and gene silencing via siRNA delivery. Our cell microscopy studies involving siRNA beacons conjugated to RNA nanoparticles demonstrate a time release study of siRNA from the RNA nanoparticles by Dicer processing. As a result of the siRNA release and incorporation into RISC, target Lamin A/C mRNA was cleaved as demonstrated in Figure 1. These experiments prove that the RNA nanoparticles do not inhibit or limit the Dicer binding, reading, or processing of siRNA because of their three-dimensional shapes. Additionally, in understanding Dicer processing of siRNA, several design principles of siRNA incorporation into RNA nanoparticles were created. First, the length of incorporated siRNA is very important, in that a minimum 22-nt sequence of the anti-sense strand is needed, with 24 nt showing the best processing efficiency by Dicer. Additionally, we have demonstrated that 2′-F modifications to the siRNA sense strand and removal of the 3′-UU overhang of the siRNA anti-sense results in reduced Dicer processing but still produces siRNA product for gene knockdown. The reduced Dicer processing of the 2′-F-modified siRNA is similar to that seen in studies by other groups looking at using 2′-F and 2′-OMethyl modifications on siRNA without nanoparticles.^89^^,^^90^ Collingwood et al. completed various modifications to both sense and anti-sense strands and overall reported reduced activities for Dicer processing.^89^ However, some modifications to siRNA have demonstrated an increased potency and even the ability to incorporate into RISC without being processed by Dicer.^90^^,^^91^ As a whole, these studies advance our understanding of the role of RNA nanoparticle design to maximize the efficiency of siRNA delivery to cancer tumor cells. Additionally, these design parameters ensure that RNA nanoparticles are stable and are able to reach tumors *in vivo*. Moving forward, these studies will be implemented.

RNA nanoparticles provide a stable *in vivo* delivery platform of RNAi, where target tissue specificity can be enhanced via conjugation of tumor-targeting ligands in addition to their rubber-like properties.^33^^,^^92^ Currently there are three platforms for delivery of siRNAs in clinical trials or approved by the FDA.^93^ These include lipid-based nanoparticles (LNPs),^94^, ^95^, ^96^ *N*-acetylgalactosamine-conjugated siRNA (GalNac-siRNA),^97^, ^98^, ^99^ and targeted RNAi Molecule (TRiM).^100^^,^^101^ These siRNA delivery platforms have become increasingly efficient at delivering siRNAs by now including targeting ligands. However, many of them typically accumulate in the liver and lungs. However, RNA nanoparticles have demonstrated their ability to avoid strong accumulation in the liver, lungs, and other healthy organs, only having strong accumulation in targeted tumors. Also, unlike other GalNac-siRNA conjugates that only target hepatocytes, RNA nanoparticles are able to harbor a variety of targeting ligands to target and accumulate in a variety of cancers including colon, breast, prostate, gastric, liver, and glioblastoma.^45^^,^^46^^,^^75^^,^^102^, ^103^, ^104^ The rubbery property, ability to deform shape under force and return to its original form upon relaxation, of RNA nanoparticles allows for a strong passive targeting effect to cancers while being able to slip through glomerular filtration and excrete to urine through the kidneys before creating toxicity to healthy organs.^92^

Additionally, RNA nanoparticles have been used to deliver dicer-substrate siRNAs to tumor sites, resulting in specific gene silencing and tumor inhibition. Cui et al. delivered *BRCAA1* siRNA to gastric cancer tumors in mice using the pRNA-3WJ labeled with folic acid.^44^ The FA conjugated onto the nanoparticle allowed for specific binding and internalization to gastric cancer cells while specifically silencing *BRCAA1*, leading to tumor growth inhibition. In a similar fashion, Lee et al. delivered luciferase siRNA as a proof of concept to glioblastoma tumors using folic acid targeting to the overexpression of FA receptors on the tumor model.^46^ Additionally, Zhang et al. utilized the pRNA-3WJ platform to deliver *MED1* siRNA to breast cancer tumors in mice to overcome tamoxifen resistance.^105^ The silencing of *MED1* by the RNA nanoparticles led to tumor inhibition as well as resensitization to tamoxifen, thus decreasing lung metastasis and cancer stem cell content. Xu et al. demonstrated the silencing of Delta-5-Desaturase (*D5D*) by pRNA-3WJ nanoparticles alongside the dihomo-γ-linolenic acid (DGLA) treatment for colon cancer suppression.^106^ Here our studies build upon this foundation of using RNA nanoparticles to deliver siRNA to cancers by examining the design properties of the substrate siRNA. Past studies have focused on incorporating 19-nt anti-sense siRNA onto 3WJ RNA nanoparticles; however, here we proved that longer 24-nt siRNA provides an increase in Dicer processing. Additionally, careful consideration must be taken for the chemical modification of the siRNA sense strand. The proven ability of RNA nanoparticles to deliver RNAi components specifically to tumors further aids in moving RNA as the third milestone in pharmaceutics following small-molecule and protein-based drugs.

### Conclusions

Through the completed studies we aimed to demonstrate the efficient processing of siRNA from RNA nanoparticles for high gene silencing while providing a comprehensive, mechanistic understanding of siRNA Dicer processing from RNA nanoparticles. As such, our pRNA-Lamin25 and 3WJ-*LUC2* beacon nanoparticles proved intercellular Dicer cleavage, RISC incorporation, and specific mRNA cleavage. Furthermore, our in-depth studies of Dicer processing of siRNA and RNA nanoparticle/siRNA conjugates not only proved that the RNA nanoparticle does not interfere in Dicer’s ability to read and process siRNA sequences but also provided design principles of incorporation of Dicer-substrate siRNA onto the nanoparticles. It can be concluded that the siRNA must be longer than 21 nt in length and allows for flexibility of chemical modifications and the inclusion of phosphate and linker nucleotides, albeit at a slight efficiency penalty. We have thus proven here, along with previous studies, that RNA nanoparticles serve as a powerful platform for the efficient delivery of siRNA.

## Materials and methods

### Preparation of RNA nanoparticles harboring siRNAs

pRNA-lamin25 nanoparticles were designed with the lamin-targeting siRNA extended off the 3′/5′ proximate end of the phi29 pRNA. The sequence of the single-stranded RNA nanoparticle is shown in Figure S3. Nanoparticles were constructed via bottom-up assembly through the construction of a dsDNA template including the T7 RNA polymerase promotor. RNA was transcribed *in vitro* by T7 RNA polymerase using previously described procedures in the Guo lab.^40^^,^^107^

Different DS/*LUC2*-siRNAs were designed to study factors affecting Dicer processing. 3WJ/*LUC2*-siRNA molecular beacon was designed to study intracellular Dicer processing. The sequences are listed in Table S1 and Table S2. Each short oligo strand to compose the 3WJs was prepared by solid-phase synthesis using an oligo-synthesizer as described previously.^102^ Molecular beacon was added to the 3WJ nanoparticles by attaching a Cy5 fluorophore to 5′-*LUC2*-antisense strand with Cy5-phosphoramidite, while BBQ650 was conjugated to 3′-3WJ-c with BBQ650-CPG column. All strands were synthesized, desalted, and purified before use.

To assemble into DS or 3WJ nanoparticles, equal molar ratios of each component strand were mixed together in 1× Tris-magnesium sulfate (TMS) buffer (50 mM Tris [pH 7.6], 100 mM NaCl, 10 mM MgCl_2_), heated to 90°C for 5 min, and slowly cooled to 37°C on a thermocycler.

### Cell culture

KB cells were cultured in RPMI-1640 medium (Life Technologies) containing 10% fetal bovine serum (FBS) in a 37°C incubator under 5% CO_2_ and a humidified atmosphere. HT29 cells were cultured in RPMI-1640 FA-deficient medium (Life Technologies) containing 10% FBS in a 37°C incubator under 5% CO_2_ and a humidified atmosphere.

### Detection of lamin A/C siRNA knockdown by 5′ RACE

100 nM pRNA-lamin25 was transfected into KB cells with FuGENE HD (Roche, Indianapolis, IN, USA). Cells were harvested 48 h after transfection. Total mRNA was isolated with the Poly(A)Purist Kit (Ambion, Austin, TX, USA). The FirstChoice RLM-RACE Kit (Ambion, Austin, TX, USA) was used in the 5′ RACE experiment, also known as single-sided PCR. With primers for the middle of the unknown sequence, mRNA is amplified in the full length, therefore either amplifying the cleaved and shortened mRNA from the siRNA or amplifying the much longer uncleaved mRNA. Thus, the PCR amplification allows for easy differentiation of cleaved product. The sequences of primers used in this study are:

Lamin gene-specific 3′ primer: 5′-CCAGTGAGTCCTCCAGGTCTCGAAG-3′
Lamin gene-specific 3′ nested primer: 5′ CCTGGCATTGTCCAGCTTGGCAGA-3′

### *In vitro* siRNA processing using Recombinant Human Dicer Enzyme

To examine the Dicer processing, 1 μg of RNA nanoparticles was incubated with 2 μL of Recombinant Human Dicer Enzyme (Genlantis), following the manufacturer’s instructions. After 6-h incubation at 37°C, 20% native PAGE was used to check the product before and after the Dicer processing. The gel was run in Tris-borate magnesium (TBM) buffer at 150 V for 1.5 h, stained with ethidium bromide (EB), and imaged on a Typhoon FLA7000 (GE) for EB and Cy5 signal.

### Stability studies of 3WJ/LUC2-siRNA nanoparticles

The serum and thermodynamic stability of 3WJ/*LUC2*-siRNA were studied with procedures previously described.^38^^,^^76^ In short, RNA nanoparticles were incubated in 10% FBS for 0–24 h and run on 15% TBM PAGE. Gel bands were quantified by ImageJ and plotted as stable RNA/total RNA. Thermodynamic stability was examined by running RNA nanoparticles in TGGE with a temperature gradient (20°C–80°C) perpendicular to the electrophoretic current. Bands were quantified by ImageJ and plotted in a similar fashion. 15% TBM PAGE was run to check the integrity and purity of the nanoparticles.

### Purification of exosomes

Exosomes were purified by a modified differential ultracentrifugation method as previously described.^30^^,^^56^^,^^78^ HEK293T cells obtained from ATCC were cultured in FiberCell Hollow Fiber Bioreactor (C2011, 20 kDa molecular weight cutoff [MWCO]) with Dulbecco's modified eagle medium (DMEM) medium with 10% exosome-free FBS. Exosome-enriched media were collected every week from the bioreactor. Exosome-enriched media were spun down at 300 rcf for 10 min to remove cells, followed by spinning at 10,000 rcf for 30 min at 4°C followed by 22-nm filtration to remove cell debris and microvesicles, and stored at −80°C until 500 mL was accumulated.

Pre-processed media were thawed slowly at 4°C overnight and then loaded into a pre-conditioned Pall Minimate tangential flow filtration (TFF) system with a 100 kDa MWCO capsule (OA100C12), preconditioned according to standard operation by the manufacturer. 500 mL of exosome-enriched medium was processed at 6 mL/min by setting pump speed at 40 mL/min. When volume reduced to ∼5 mL, 200 mL of sterile Dulbecco's phosphate buffered saline (DPBS) was added as a washing step, the system continued to run until the volume reduced to ∼5 mL again, and medium was then collected. Two 15-mL DPBS wash steps were performed, and a total 30-mL wash was collected and combined with the 5-mL sample from the last step.

The 35-mL post-TFF media were then further purified by 100,000 rcf ultracentrifugation using a SW28 rotor (Beckman Coulter) for 90 min at 4°C. 200 μL of 60% iodixanol (Sigma) was added to the bottom of each tube to serve as iso-osmotic cushion as previously reported.^30^^,^^56^^,^^78^ The supernatant was carefully removed from the top, and ∼2 mL of the fraction close to the interface and cushion was collected.

### Exosome characterization

Nanoparticle tracking analysis (NTA) was carried out with the Malvern NanoSight NS300 system on exosomes re-suspended in phosphate buffered saline (PBS) at a concentration of 10 μg of protein per milliliter for analysis following published methods.^30^ Three 10-s videos recorded all events for further analysis by NTA software. The Brownian motion of each particle is tracked between frames, ultimately allowing for calculation of the size through application of the Stokes–Einstein equation.

Purified exosomes were assayed for exosomal protein markers via western blot. Exosomes were loaded onto TDX FastCast SDS-PAGE gels (Bio-Rad, Hercules, CA, USA), run at 100 V for 2 h, and transferred from gel to membrane. Membranes were blocked by 5% fat-free milk at room temperature for 1 h and incubated overnight in primary antibody. Protein bands were detected with an ECL system (Pierce) after incubating in the horseradish peroxidase (HRP)-conjugated secondary antibody for 1 h at room temperature and exposed to film for autoradiography. Primary antibodies used for western blot analysis were rabbit anti-human TSG101 (Thermo Scientific, PA5-31260), rabbit anti-human integrin α4 (Cell Signaling, 4711S), rabbit anti-human integrin α6 (Cell Signaling, 3750S), rabbit anti-human integrin β1 (Cell Signaling, 4706S), rabbit anti-human integrin β4 (Cell Signaling, 4707S), rabbit anti-human integrin β5 (Cell Signaling, 4708S), and rabbit anti-human Glypican 1 (Thermo Fisher, PA5-28055).

### Loading of 3WJ/LUC2-siRNA and 3WJ/siSur into FA/EV and EGFR/EV

Exosomes (EVs) (100 μg of total protein) and RNA nanoparticles harboring *LUC2* siRNA or Survivin siRNA (10 μg) were mixed in 100 μL of PBS with 10 μL of Exo-Fect Exosome transfection solution (System Biosciences), followed by a heat-shock protocol to complete the RNA nanoparticle loading. Cholesterol-modified 3WJ/FA or cholesterol-modified 3WJ/EGFR (or 3WJ) nanoparticles were incubated with RNA-loaded exosomes for decoration at an average ratio of 5,000:1 (RNA:exosome) at 37°C for 45 min and then left on ice for 1 h to prepare the FA/EV and EGFR/EV. To purify RNA-decorated EVs, 400 μL of RNA-decorated EVs was washed with 5 mL of PBS in a SW-55 tube that contained 20 μL of 60% iodixanol cushion and spun at 100,000 × *g* for 70 min at 4°C. All the pellets in the cushion were collected and suspended in 400 μL of sterile PBS for further use.

### Binding and internalization assay of FA/Exo/Luc2-siRNA

For cell binding assays, HT29 cells were incubated with FA/EV/*LUC2*-siRNA/Cy5, 3WJ/EV/*LUC2*-siRNA/Cy5, 3WJ/FA/*LUC2*-siRNA/Cy5, and 3WJ/*LUC2*-siRNA/Cy5 at 37°C for 1 h before flow cytometry. Samples were incubated at a concentration of 100 nM of the Cy5 RNA strand.

To study internalization, FA/EV/*LUC2*-siRNA/Cy5 as well as control groups including 3WJ/EV/*LUC2*-siRNA/Cy5 and EV/*LUC2*-siRNA/Cy5 were incubated with HT29 cells at 37°C for 1 h. Samples were incubated at a concentration of 100 nM of the Cy5 RNA strand. After washing with PBS, the cells were fixed by 4% paraformaldehyde and stained by Alexa Fluor 488 phalloidin (Invitrogen) according to manufacturer’s instructions and DAPI for cell nucleus staining and mounted with ProLong Gold Antifade Reagent (Life Technologies, Carlsbad, CA, USA). The cells were then analyzed for binding and cell entry by an Olympus FV3000 Confocal System microscope.

### Intracellular imaging of siRNA processing from 3WJ via molecular beacon

To monitor intracellular Dicer processing, RNA nanoparticles (3WJ/*LUC2*-siRNA-tru beacon, 3WJ/*LUC2*-siRNA beacon, 3WJ/*LUC2*-siRNA Cy5) were loaded to exosomes, respectively, further decorated by FA as described above. After incubation with HT29 cells for 0.5, 2, and 4 h, cells were fixed and stained as described above. Samples were incubated at a concentration of 100 nM of the Cy5 RNA strand. Cy5 signal was monitored for each group at different time points by confocal microscopy under the same parameters.

## Supplemental information

Document 1. Figures S1–S3 and Tables S1 and S2

Document 2. Article plus supplemental information

## References

[1] Agrawal N., Dasaradhi P.V., Mohmmed A., Malhotra P., Bhatnagar R.K., Mukherjee S.K. (2003). RNA interference: biology, mechanism, and applications. Microbiol. Mol. Biol. Rev..

[2] Bernstein E., Caudy A.A., Hammond S.M., Hannon G.J. (2001). Role for a bidentate ribonuclease in the initiation step of RNA interference. Nature.

[3] Lam J.K., Chow M.Y., Zhang Y., Leung S.W. (2015). siRNA Versus miRNA as Therapeutics for Gene Silencing. Mol. Ther. Nucleic Acids.

[4] Couzin J. (2002). Breakthrough of the year. Small RNAs make big splash. Science.

[5] Fire A., Xu S., Montgomery M.K., Kostas S.A., Driver S.E., Mello C.C. (1998). Potent and specific genetic interference by double-stranded RNA in Caenorhabditis elegans. Nature.

[6] Elbashir S.M., Harborth J., Lendeckel W., Yalcin A., Weber K., Tuschl T. (2001). Duplexes of 21-nucleotide RNAs mediate RNA interference in cultured mammalian cells. Nature.

[7] Hoy S.M. (2018). Patisiran: First Global Approval. Drugs.

[8] Urits I., Swanson D., Swett M.C., Patel A., Berardino K., Amgalan A., Berger A.A., Kassem H., Kaye A.D., Viswanath O. (2020). A Review of Patisiran (ONPATTRO®) for the Treatment of Polyneuropathy in People with Hereditary Transthyretin Amyloidosis. Neurol. Ther..

[9] Gomá-Garcés E., Pérez-Gómez M.V., Ortíz A. (2020). Givosiran for Acute Intermittent Porphyria. N. Engl. J. Med..

[10] Scott L.J. (2020). Givosiran: First Approval. Drugs.

[11] Scott L.J., Keam S.J. (2021). Lumasiran: First Approval. Drugs.

[12] Shu Y., Pi F., Sharma A., Rajabi M., Haque F., Shu D., Leggas M., Evers B.M., Guo P. (2014). Stable RNA nanoparticles as potential new generation drugs for cancer therapy. Adv. Drug Deliv. Rev..

[13] Wang H.W., Noland C., Siridechadilok B., Taylor D.W., Ma E., Felderer K., Doudna J.A., Nogales E. (2009). Structural insights into RNA processing by the human RISC-loading complex. Nat. Struct. Mol. Biol..

[14] Raja M.A.G., Katas H., Amjad M.W. (2019). Design, mechanism, delivery and therapeutics of canonical and Dicer-substrate siRNA. Asian J Pharm Sci.

[15] Gregory R.I., Chendrimada T.P., Cooch N., Shiekhattar R. (2005). Human RISC couples microRNA biogenesis and posttranscriptional gene silencing. Cell.

[16] Kim D.H., Behlke M.A., Rose S.D., Chang M.S., Choi S., Rossi J.J. (2005). Synthetic dsRNA Dicer substrates enhance RNAi potency and efficacy. Nat. Biotechnol..

[17] Hoerter J.A., Walter N.G. (2007). Chemical modification resolves the asymmetry of siRNA strand degradation in human blood serum. RNA.

[18] Watts J.K., Deleavey G.F., Damha M.J. (2008). Chemically modified siRNA: tools and applications. Drug Discov. Today.

[19] Bramsen J.B., Kjems J. (2011). Chemical modification of small interfering RNA. Methods Mol. Biol..

[20] Jackson A.L., Burchard J., Leake D., Reynolds A., Schelter J., Guo J., Johnson J.M., Lim L., Karpilow J., Nichols K. (2006). Position-specific chemical modification of siRNAs reduces “off-target” transcript silencing. RNA.

[21] Selvam C., Mutisya D., Prakash S., Ranganna K., Thilagavathi R. (2017). Therapeutic potential of chemically modified siRNA: Recent trends. Chem. Biol. Drug Des..

[22] Lau P.W., Guiley K.Z., De N., Potter C.S., Carragher B., MacRae I.J. (2012). The molecular architecture of human Dicer. Nat. Struct. Mol. Biol..

[23] Sashital D.G., Doudna J.A. (2010). Structural insights into RNA interference. Curr. Opin. Struct. Biol..

[24] Song J.J., Liu J., Tolia N.H., Schneiderman J., Smith S.K., Martienssen R.A., Hannon G.J., Joshua-Tor L. (2003). The crystal structure of the Argonaute2 PAZ domain reveals an RNA binding motif in RNAi effector complexes. Nat. Struct. Biol..

[25] Podolska K., Sedlak D., Bartunek P., Svoboda P. (2014). Fluorescence-based high-throughput screening of dicer cleavage activity. J. Biomol. Screen..

[26] Harborth J., Elbashir S.M., Vandenburgh K., Manninga H., Scaringe S.A., Weber K., Tuschl T. (2003). Sequence, chemical, and structural variation of small interfering RNAs and short hairpin RNAs and the effect on mammalian gene silencing. Antisense Nucleic Acid Drug Dev..

[27] MacRae I.J., Zhou K., Doudna J.A. (2007). Structural determinants of RNA recognition and cleavage by Dicer. Nat. Struct. Mol. Biol..

[28] Park J.E., Heo I., Tian Y., Simanshu D.K., Chang H., Jee D., Patel D.J., Kim V.N. (2011). Dicer recognizes the 5′ end of RNA for efficient and accurate processing. Nature.

[29] Vermeulen A., Behlen L., Reynolds A., Wolfson A., Marshall W.S., Karpilow J., Khvorova A. (2005). The contributions of dsRNA structure to Dicer specificity and efficiency. RNA.

[30] Pi F., Binzel D.W., Lee T.J., Li Z., Sun M., Rychahou P., Li H., Haque F., Wang S., Croce C.M. (2018). Nanoparticle orientation to control RNA loading and ligand display on extracellular vesicles for cancer regression. Nat. Nanotechnol..

[31] Guo P., Coban O., Snead N.M., Trebley J., Hoeprich S., Guo S., Shu Y. (2010). Engineering RNA for targeted siRNA delivery and medical application. Adv. Drug Deliv. Rev..

[32] Jasinski D., Haque F., Binzel D.W., Guo P. (2017). Advancement of the Emerging Field of RNA Nanotechnology. ACS Nano.

[33] Xu C., Haque F., Jasinski D.L., Binzel D.W., Shu D., Guo P. (2018). Favorable biodistribution, specific targeting and conditional endosomal escape of RNA nanoparticles in cancer therapy. Cancer Lett..

[34] Guo P. (2010). The emerging field of RNA nanotechnology. Nat. Nanotechnol..

[35] Guo P., Zhang C., Chen C., Garver K., Trottier M. (1998). Inter-RNA interaction of phage phi29 pRNA to form a hexameric complex for viral DNA transportation. Mol. Cell.

[36] Shu D., Shu Y., Haque F., Abdelmawla S., Guo P. (2011). Thermodynamically stable RNA three-way junction for constructing multifunctional nanoparticles for delivery of therapeutics. Nat. Nanotechnol..

[37] Khisamutdinov E.F., Jasinski D.L., Guo P. (2014). RNA as a boiling-resistant anionic polymer material to build robust structures with defined shape and stoichiometry. ACS Nano.

[38] Piao X., Wang H., Binzel D.W., Guo P. (2018). Assessment and comparison of thermal stability of phosphorothioate-DNA, DNA, RNA, 2′-F RNA, and LNA in the context of Phi29 pRNA 3WJ. RNA.

[39] Haque F., Shu D., Shu Y., Shlyakhtenko L.S., Rychahou P.G., Evers B.M., Guo P. (2012). Ultrastable synergistic tetravalent RNA nanoparticles for targeting to cancers. Nano Today.

[40] Shu Y., Haque F., Shu D., Li W., Zhu Z., Kotb M., Lyubchenko Y., Guo P. (2013). Fabrication of 14 different RNA nanoparticles for specific tumor targeting without accumulation in normal organs. RNA.

[41] Liu J., Guo S., Cinier M., Shlyakhtenko L.S., Shu Y., Chen C., Shen G., Guo P. (2011). Fabrication of stable and RNase-resistant RNA nanoparticles active in gearing the nanomotors for viral DNA packaging. ACS Nano.

[42] Abdelmawla S., Guo S., Zhang L., Pulukuri S.M., Patankar P., Conley P., Trebley J., Guo P., Li Q.X. (2011). Pharmacological characterization of chemically synthesized monomeric phi29 pRNA nanoparticles for systemic delivery. Mol. Ther..

[43] Shu D., Khisamutdinov E.F., Zhang L., Guo P. (2014). Programmable folding of fusion RNA in vivo and in vitro driven by pRNA 3WJ motif of phi29 DNA packaging motor. Nucleic Acids Res..

[44] Cui D., Zhang C., Liu B., Shu Y., Du T., Shu D., Wang K., Dai F., Liu Y., Li C. (2015). Regression of Gastric Cancer by Systemic Injection of RNA Nanoparticles Carrying both Ligand and siRNA. Sci. Rep..

[45] Rychahou P., Haque F., Shu Y., Zaytseva Y., Weiss H.L., Lee E.Y., Mustain W., Valentino J., Guo P., Evers B.M. (2015). Delivery of RNA nanoparticles into colorectal cancer metastases following systemic administration. ACS Nano.

[46] Lee T.J., Haque F., Shu D., Yoo J.Y., Li H., Yokel R.A., Horbinski C., Kim T.H., Kim S.H., Kwon C.H. (2015). RNA nanoparticle as a vector for targeted siRNA delivery into glioblastoma mouse model. Oncotarget.

[47] El Andaloussi S., Mager I., Breakefield X.O., Wood M.J.A. (2013). Extracellular vesicles: biology and emerging therapeutic opportunities. Nat. Rev. Drug Discov..

[48] Valadi H., Ekström K., Bossios A., Sjöstrand M., Lee J.J., Lötvall J.O. (2007). Exosome-mediated transfer of mRNAs and microRNAs is a novel mechanism of genetic exchange between cells. Nat. Cell Biol..

[49] Alvarez-Erviti L., Seow Y., Yin H., Betts C., Lakhal S., Wood M.J. (2011). Delivery of siRNA to the mouse brain by systemic injection of targeted exosomes. Nat. Biotechnol..

[50] Ohno S., Takanashi M., Sudo K., Ueda S., Ishikawa A., Matsuyama N., Fujita K., Mizutani T., Ohgi T., Ochiya T. (2013). Systemically injected exosomes targeted to EGFR deliver antitumor microRNA to breast cancer cells. Mol. Ther..

[51] Shtam T.A., Kovalev R.A., Varfolomeeva E.Y., Makarov E.M., Kil Y.V., Filatov M.V. (2013). Exosomes are natural carriers of exogenous siRNA to human cells in vitro. Cell Commun. Signal..

[52] van Dommelen S.M., Vader P., Lakhal S., Kooijmans S.A., van Solinge W.W., Wood M.J., Schiffelers R.M. (2012). Microvesicles and exosomes: opportunities for cell-derived membrane vesicles in drug delivery. J. Control. Release.

[53] Al-Nedawi K., Meehan B., Micallef J., Lhotak V., May L., Guha A., Rak J. (2008). Intercellular transfer of the oncogenic receptor EGFRvIII by microvesicles derived from tumour cells. Nat. Cell Biol..

[54] Skog J., Würdinger T., van Rijn S., Meijer D.H., Gainche L., Sena-Esteves M., Curry W.T., Carter B.S., Krichevsky A.M., Breakefield X.O. (2008). Glioblastoma microvesicles transport RNA and proteins that promote tumour growth and provide diagnostic biomarkers. Nat. Cell Biol..

[55] El Andaloussi S., Lakhal S., Mäger I., Wood M.J. (2013). Exosomes for targeted siRNA delivery across biological barriers. Adv. Drug Deliv. Rev..

[56] Zheng Z., Li Z., Xu C., Guo B., Guo P. (2019). Folate-displaying exosome mediated cytosolic delivery of siRNA avoiding endosome trapping. J. Control. Release.

[57] Afonin K.A., Cieply D.J., Leontis N.B. (2008). Specific RNA self-assembly with minimal paranemic motifs. J. Am. Chem. Soc..

[58] Chworos A., Severcan I., Koyfman A.Y., Weinkam P., Oroudjev E., Hansma H.G., Jaeger L. (2004). Building programmable jigsaw puzzles with RNA. Science.

[59] Geary C., Rothemund P.W., Andersen E.S. (2014). A single-stranded architecture for cotranscriptional folding of RNA nanostructures. Science.

[60] Grabow W.W., Jaeger L. (2014). RNA self-assembly and RNA nanotechnology. Acc. Chem. Res..

[61] Hansma H.G., Oroudjev E., Baudrey S., Jaeger L. (2003). TectoRNA and ‘kissing-loop’ RNA: atomic force microscopy of self-assembling RNA structures. J. Microsc..

[62] Hao C., Li X., Tian C., Jiang W., Wang G., Mao C. (2014). Construction of RNA nanocages by re-engineering the packaging RNA of Phi29 bacteriophage. Nat. Commun..

[63] Jaeger L., Westhof E., Leontis N.B. (2001). TectoRNA: modular assembly units for the construction of RNA nano-objects. Nucleic Acids Res..

[64] Ohno H., Saito H. (2016). RNA and RNP as Building Blocks for Nanotechnology and Synthetic Biology. Prog. Mol. Biol. Transl. Sci..

[65] Jedrzejczyk D., Chworos A. (2019). Self-Assembling RNA Nanoparticle for Gene Expression Regulation in a Model System. ACS Synth. Biol..

[66] Afonin K.A., Kireeva M., Grabow W.W., Kashlev M., Jaeger L., Shapiro B.A. (2012). Co-transcriptional assembly of chemically modified RNA nanoparticles functionalized with siRNAs. Nano Lett..

[67] Stewart J.M., Viard M., Subramanian H.K., Roark B.K., Afonin K.A., Franco E. (2016). Programmable RNA microstructures for coordinated delivery of siRNAs. Nanoscale.

[68] Zakrevsky P., Kasprzak W.K., Heinz W.F., Wu W., Khant H., Bindewald E., Dorjsuren N., Fields E.A., de Val N., Jaeger L., Shapiro B.A. (2020). Truncated tetrahedral RNA nanostructures exhibit enhanced features for delivery of RNAi substrates. Nanoscale.

[69] Rackley L., Stewart J.M., Salotti J., Krokhotin A., Shah A., Halman J.R., Juneja R., Smollett J., Lee L., Roark K. (2018). RNA Fibers as Optimized Nanoscaffolds for siRNA Coordination and Reduced Immunological Recognition. Adv. Funct. Mater..

[70] Lee J.B., Hong J., Bonner D.K., Poon Z., Hammond P.T. (2012). Self-assembled RNA interference microsponges for efficient siRNA delivery. Nat. Mater..

[71] Halman J.R., Kim K.T., Gwak S.J., Pace R., Johnson M.B., Chandler M.R., Rackley L., Viard M., Marriott I., Lee J.S., Afonin K.A. (2020). A cationic amphiphilic co-polymer as a carrier of nucleic acid nanoparticles (Nanps) for controlled gene silencing, immunostimulation, and biodistribution. Nanomedicine (Lond.).

[72] Nordmeier S., Ke W., Afonin K.A., Portnoy V. (2020). Exosome mediated delivery of functional nucleic acid nanoparticles (NANPs). Nanomedicine (Lond.).

[73] Frohman M.A., Dush M.K., Martin G.R. (1988). Rapid production of full-length cDNAs from rare transcripts: amplification using a single gene-specific oligonucleotide primer. Proc. Natl. Acad. Sci. USA.

[74] Zhang H.M., Su Y., Guo S., Yuan J., Lim T., Liu J., Guo P., Yang D. (2009). Targeted delivery of anti-coxsackievirus siRNAs using ligand-conjugated packaging RNAs. Antiviral Res..

[75] Binzel D.W., Shu Y., Li H., Sun M., Zhang Q., Shu D., Guo B., Guo P. (2016). Specific Delivery of MiRNA for High Efficient Inhibition of Prostate Cancer by RNA Nanotechnology. Mol. Ther..

[76] Binzel D.W., Khisamutdinov E.F., Guo P. (2014). Entropy-driven one-step formation of Phi29 pRNA 3WJ from three RNA fragments. Biochemistry.

[77] Harding C., Stahl P. (1983). Transferrin recycling in reticulocytes: pH and iron are important determinants of ligand binding and processing. Biochem. Biophys. Res. Commun..

[78] Li Z., Wang H., Yin H., Bennett C., Zhang H.G., Guo P. (2018). Arrowtail RNA for Ligand Display on Ginger Exosome-like Nanovesicles to Systemic Deliver siRNA for Cancer Suppression. Sci. Rep..

[79] Gu S., Jin L., Zhang Y., Huang Y., Zhang F., Valdmanis P.N., Kay M.A. (2012). The loop position of shRNAs and pre-miRNAs is critical for the accuracy of dicer processing in vivo. Cell.

[80] Lau P.W., Potter C.S., Carragher B., MacRae I.J. (2009). Structure of the human Dicer-TRBP complex by electron microscopy. Structure.

[81] Li Z., Yang L., Wang H., Binzel D.W., Williams T.M., Guo P. (2021). Non-Small-Cell Lung Cancer Regression by siRNA Delivered Through Exosomes That Display EGFR RNA Aptamer. Nucleic Acid Ther..

[82] Charbe N.B., Amnerkar N.D., Ramesh B., Tambuwala M.M., Bakshi H.A., Aljabali A.A.A., Khadse S.C., Satheeshkumar R., Satija S., Metha M. (2020). Small interfering RNA for cancer treatment: overcoming hurdles in delivery. Acta Pharm. Sin. B.

[83] Editorial (2006). A billion dollar punt. Nat. Biotechnol..

[84] Duchaine T.F., Slack F.J. (2009). RNA interference and micro RNA-oriented therapy in cancer: rationales, promises, and challenges. Curr. Oncol..

[85] Couzin-Frankel J. (2010). Drug research. Roche exits RNAi field, cuts 4800 jobs. Science.

[86] Huggett B., Paisner K. (2017). The commercial tipping point. Nat. Biotechnol..

[87] Haussecker D. (2008). The business of RNAi therapeutics. Hum. Gene Ther..

[88] Morrison C. (2018). Alnylam prepares to land first RNAi drug approval. Nat. Rev. Drug Discov..

[89] Collingwood M.A., Rose S.D., Huang L., Hillier C., Amarzguioui M., Wiiger M.T., Soifer H.S., Rossi J.J., Behlke M.A. (2008). Chemical modification patterns compatible with high potency dicer-substrate small interfering RNAs. Oligonucleotides.

[90] Foster D.J., Barros S., Duncan R., Shaikh S., Cantley W., Dell A., Bulgakova E., O’Shea J., Taneja N., Kuchimanchi S. (2012). Comprehensive evaluation of canonical versus Dicer-substrate siRNA in vitro and in vivo. RNA.

[91] Salomon W., Bulock K., Lapierre J., Pavco P., Woolf T., Kamens J. (2010). Modified dsRNAs that are not processed by Dicer maintain potency and are incorporated into the RISC. Nucleic Acids Res..

[92] Ghimire C., Wang H., Li H., Vieweger M., Xu C., Guo P. (2020). RNA Nanoparticles as Rubber for Compelling Vessel Extravasation to Enhance Tumor Targeting and for Fast Renal Excretion to Reduce Toxicity. ACS Nano.

[93] Saw P.E., Song E.W. (2020). siRNA therapeutics: a clinical reality. Sci. China Life Sci..

[94] Yonezawa S., Koide H., Asai T. (2020). Recent advances in siRNA delivery mediated by lipid-based nanoparticles. Adv. Drug Deliv. Rev..

[95] Lin Q., Chen J., Zhang Z., Zheng G. (2014). Lipid-based nanoparticles in the systemic delivery of siRNA. Nanomedicine (Lond.).

[96] Li T., Huang L., Yang M. (2020). Lipid-based Vehicles for siRNA Delivery in Biomedical Field. Curr. Pharm. Biotechnol..

[97] Nair J.K., Attarwala H., Sehgal A., Wang Q., Aluri K., Zhang X., Gao M., Liu J., Indrakanti R., Schofield S. (2017). Impact of enhanced metabolic stability on pharmacokinetics and pharmacodynamics of GalNAc-siRNA conjugates. Nucleic Acids Res..

[98] Foster D.J., Brown C.R., Shaikh S., Trapp C., Schlegel M.K., Qian K., Sehgal A., Rajeev K.G., Jadhav V., Manoharan M. (2018). Advanced siRNA Designs Further Improve In Vivo Performance of GalNAc-siRNA Conjugates. Mol. Ther..

[99] Springer A.D., Dowdy S.F. (2018). GalNAc-siRNA Conjugates: Leading the Way for Delivery of RNAi Therapeutics. Nucleic Acid Ther..

[100] Turner A.M., Stolk J., Bals R., Lickliter J.D., Hamilton J., Christianson D.R., Given B.D., Burdon J.G., Loomba R., Stoller J.K., Teckman J.H. (2018). Hepatic-targeted RNA interference provides robust and persistent knockdown of alpha-1 antitrypsin levels in ZZ patients. J. Hepatol..

[101] Sebestyén M.G., Wong S.C., Trubetskoy V., Lewis D.L., Wooddell C.I. (2015). Targeted in vivo delivery of siRNA and an endosome-releasing agent to hepatocytes. Methods Mol. Biol..

[102] Guo S., Vieweger M., Zhang K., Yin H., Wang H., Li X., Li S., Hu S., Sparreboom A., Evers B.M. (2020). Ultra-thermostable RNA nanoparticles for solubilizing and high-yield loading of paclitaxel for breast cancer therapy. Nat. Commun..

[103] Shu D., Li H., Shu Y., Xiong G., Carson W.E., Haque F., Xu R., Guo P. (2015). Systemic Delivery of Anti-miRNA for Suppression of Triple Negative Breast Cancer Utilizing RNA Nanotechnology. ACS Nano.

[104] Wang H., Ellipilli S., Lee W.J., Li X., Vieweger M., Ho Y.S., Guo P. (2021). Multivalent rubber-like RNA nanoparticles for targeted co-delivery of paclitaxel and MiRNA to silence the drug efflux transporter and liver cancer drug resistance. J. Control. Release.

[105] Zhang Y., Leonard M., Shu Y., Yang Y., Shu D., Guo P., Zhang X. (2017). Overcoming Tamoxifen Resistance of Human Breast Cancer by Targeted Gene Silencing Using Multifunctional pRNA Nanoparticles. ACS Nano.

[106] Xu Y., Pang L., Wang H., Xu C., Shah H., Guo P., Shu D., Qian S.Y. (2019). Specific delivery of delta-5-desaturase siRNA via RNA nanoparticles supplemented with dihomo-γ-linolenic acid for colon cancer suppression. Redox Biol..

[107] Shu Y., Shu D., Haque F., Guo P. (2013). Fabrication of pRNA nanoparticles to deliver therapeutic RNAs and bioactive compounds into tumor cells. Nat. Protoc..

