# Supplementary material for: Rational design for controlled release of Dicer-substrate siRNA harbored in phi29 pRNA-based nanoparticles
Source: Mol Ther Nucleic Acids. 2021 Aug 8;25:524–35. doi: 10.1016/j.omtn.2021.07.021 (PMC8463318; doi:10.1016/j.omtn.2021.07.021)
Supplement: Document 1. Figures S1–S3 and Tables S1 and S2 [file mmc1.pdf]

OMTN, Volume *25*

Supplemental information

Rational design for controlled release of Dicer-substrate siRNA harbored in phi29 pRNA-based nanoparticles

Daniel W. Binzel, Songchuan Guo, Hongran Yin, Tae Jin Lee, Shujun Liu, Dan Shu, and Peixuan Guo

Supplemental Materials

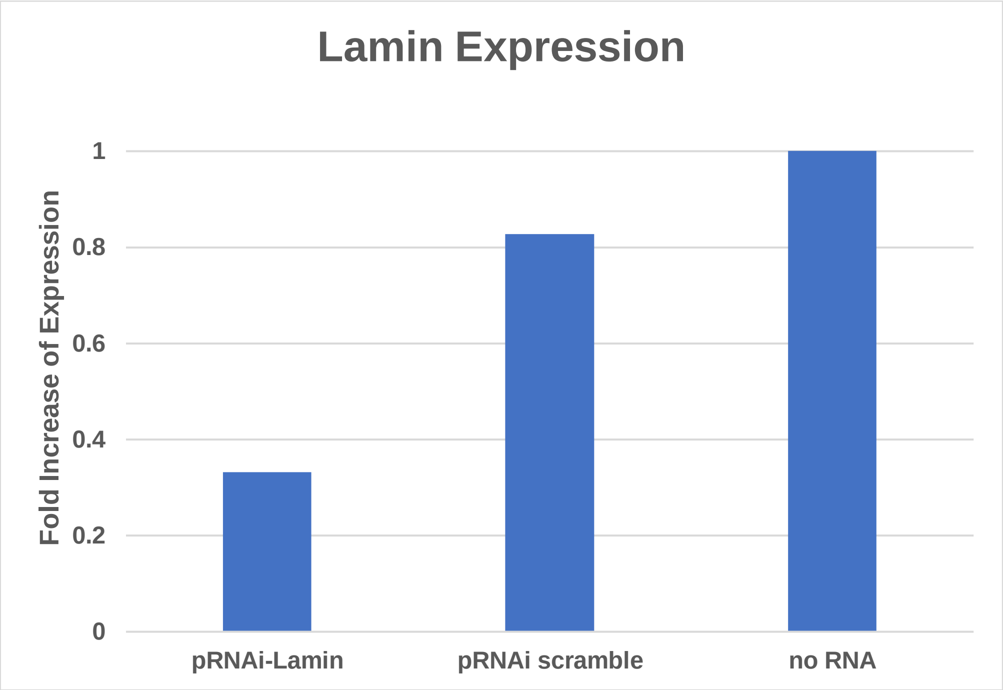

Figure S1: Quantitative qRT-PCR of Lamin expression. Lamin expression was assayed following incubation with pRNAi nanoparticles with Lamin siRNA and scrambled siRNA in KB cells. The data were analyzed by the comparative CT Method (ΔΔCT Method).

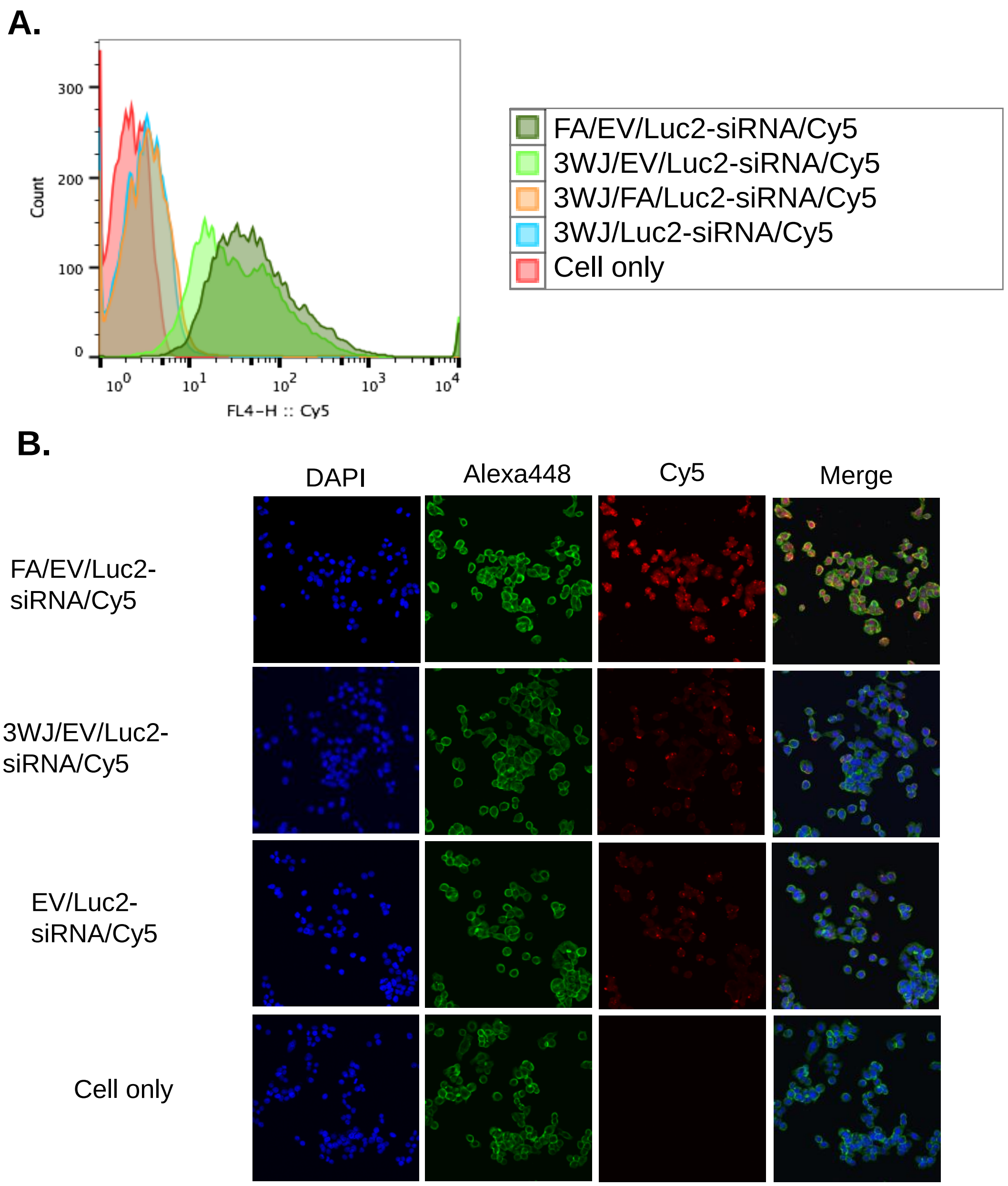

Figure S2: Binding and internalization of FA/EV carrying 3WJ/Luc2-siRNA. A. Binding of FA/EV/Luc2-siRNA and control groups to HT29 cells by Flow Cytometry. **B.** Images taken by confocal microscopy for internalization study (Blue: nuclei; Green: Cytoskeleton; Red: RNA).

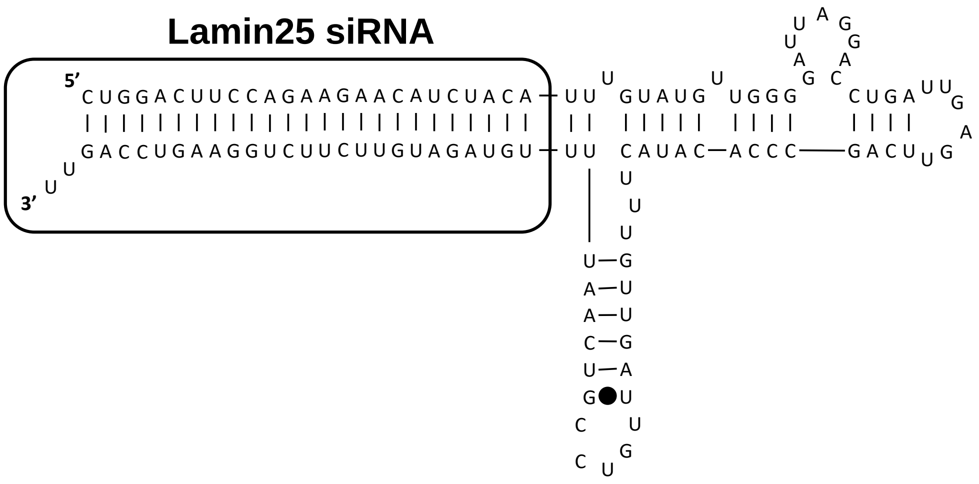

pRNA-Lamin25 siRNA sequence:

5’-CUG GAC UUC CAG AAG AAC AUC UAC AUU UGU AUG UUG GGG AUU AGG ACC UGA UUG AGU UCA GCC CAC AUA CUU UGU UGA UUG UCC GUC AAU UUU GUA GAU GUU CUU CUG GAA GUC CAG UU-3’

Figure S3: Design of pRNA-Lamin25 nanoparticle. Secondary structuring and sequence of pRNA-Lamin25 RNA nanoparticles. Blue letter denoting the siRNA sequence. Design is based on Zhang *et al. Antiviral Res* 2009, 83:307-16.[1]

Table S1: Luc2 siRNA sequences.

19 bp siRNA	
Sense	5’- pCUUACGCUGAGUACUUCGA-3’
Anti-sense	3’-UUGAAUGCGACUCAUGAAGCUp-5’
21 bp siRNA	
Sense	5’- pCUUACGCUGAGUACUUCGAUU-3’
Anti-sense	3’-UUGAAUGCGACUCAUGAAGCUAAp-5’
22 bp siRNA	
Sense	5’- pCUUACGCUGAGUACUUCGAUUU-3’
Anti-sense	3’-UUGAAUGCGACUCAUGAAGCUAAAp-5’
23 bp siRNA	
Sense	5’- pCUUACGCUGAGUACUUCGAUUUU-3’
Anti-sense	3’-UUGAAUGCGACUCAUGAAGCUAAAAp-5’
24 bp siRNA	
Sense	5’- pCUUACGCUGAGUACUUCGAUUUUC-3’
Anti-sense	3’-UUGAAUGCGACUCAUGAAGCUAAAAGp-5’

*p denotes 5’-phosphate

Table S2: 3WJ sequences.

3WJ-a	5’-UUGCCAUGUGUAUGUGGG-3’
3WJ-b	5’-CCCACAUACUUUGUUGAUCC-3’
3WJ-c	5’-GGAUCAAUCAUGGCAA-3’
3WJ-a/Luc2 sense siRNA	5’-CUUACGCUGAGUACUUCGAUUUUCUUGCCAUGUGUAUGUGGG-3’
3WJ-a/Luc2 sense truncated	5’-CUUACGCUGAGUACUUCGAUUGCCAUGUGUAUGUGGG-3’
3WJ-c/BBQ	5’-GGAUCAAUCAUGGCAA/BBQ-3’

Supplemental References

1. Zhang, HM, Su, Y, Guo, S, Yuan, J, Lim, T, Liu, J, Guo, P, and Yang, D (2009). Targeted delivery of anti-coxsackievirus siRNAs using ligand-conjugated packaging RNAs. *Antiviral Res* **83**: 307-316.
